# Allocating HIV Prevention Funds in the United States: Recommendations from an Optimization Model

**DOI:** 10.1371/journal.pone.0037545

**Published:** 2012-06-06

**Authors:** Arielle Lasry, Stephanie L. Sansom, Katherine A. Hicks, Vladislav Uzunangelov

**Affiliations:** 1 Division of HIV/AIDS Prevention, Centers for Disease Control and Prevention, Atlanta, Georgia, United States of America; 2 RTI International, Research Triangle Park, North Carolina, United States of America; UNAIDS, Switzerland

## Abstract

The Centers for Disease Control and Prevention (CDC) had an annual budget of approximately $327 million to fund health departments and community-based organizations for core HIV testing and prevention programs domestically between 2001 and 2006. Annual HIV incidence has been relatively stable since the year 2000 [1] and was estimated at 48,600 cases in 2006 and 48,100 in 2009 [2]. Using estimates on HIV incidence, prevalence, prevention program costs and benefits, and current spending, we created an HIV resource allocation model that can generate a mathematically optimal allocation of the Division of HIV/AIDS Prevention’s extramural budget for HIV testing, and counseling and education programs. The model’s data inputs and methods were reviewed by subject matter experts internal and external to the CDC via an extensive validation process. The model projects the HIV epidemic for the United States under different allocation strategies under a fixed budget. Our objective is to support national HIV prevention planning efforts and inform the decision-making process for HIV resource allocation. Model results can be summarized into three main recommendations. First, more funds should be allocated to testing and these should further target men who have sex with men and injecting drug users. Second, counseling and education interventions ought to provide a greater focus on HIV positive persons who are aware of their status. And lastly, interventions should target those at high risk for transmitting or acquiring HIV, rather than lower-risk members of the general population. The main conclusions of the HIV resource allocation model have played a role in the introduction of new programs and provide valuable guidance to target resources and improve the impact of HIV prevention efforts in the United States.

## Introduction

At the end of 2006, HIV prevalence among the adult and adolescent population in the United States was estimated at 1.1 million, with 21% unaware of their seropositivity [3]. A 2008 survey of 21 cities indicated that 19% of men who have sex with men (MSM) were HIV infected and 44% of those were unaware of their infection [4]. Annual HIV incidence has been relatively stable since the year 2000 [1] and was estimated at 48,600 cases in 2006 and 48,100 in 2009 [2]. More than half (56%) of these cases occurred among MSM, while injection drug use and heterosexual contact accounted for 11% and 29% of incident cases, respectively in 2006 [2]. Black and Hispanic populations in the US are disproportionately affected by HIV. In 2006, 44% and 18% of new HIV infections were among black individuals and Hispanics, respectively, while these populations represent 13% and 15% of the general US adult population, respectively [2].

Approximately 84% of all federal funding for domestic HIV prevention is channeled through the Division of HIV/AIDS Prevention (DHAP) at the Centers for Disease Control and Prevention (CDC) [5]. In 2006, DHAP had an extramural budget of approximately $650 million; of that, approximately $327 million was used to fund health departments and community-based organizations for core HIV testing and prevention programs domestically.

Using the best and most current available estimates on HIV incidence, prevalence, prevention program costs and benefits, and current spending, we created an HIV resource allocation model that can generate the mathematically optimal allocation of DHAP’s budget. We explore the allocation of DHAP’s budget and therefore focus specifically on the main prevention programs currently funded by DHAP, HIV testing, and counseling and education programs. Evidence supporting biomedical measures such as circumcision, pre-exposure prophylaxis (PrEP) and antiretroviral therapy (ART) for HIV prevention has recently been released [6], [7], [8]. However, medical services and funding for treatment are not allowable expenses for CDC’s HIV prevention funds, and HIV care and treatment is administered by other federal agencies. The model projects the HIV epidemic for the United States under different allocation strategies of a fixed budget. It selects the allocation scenario that minimizes incidence over a five-year time horizon. Our objective was to support DHAP’s planning efforts and inform the decision-making process for HIV resource allocation.

## Methods

The HIV resource allocation model is comprised of two components that interact: an epidemic model that projects HIV infections over time given a specific funding allocation scenario, and an optimization model that supplies different allocation scenarios to the epidemic model, stopping with the allocation that yields the fewest new infections.

### The Epidemic Model

The epidemic model is a dynamic compartmental model defined by a set of difference equations. Details of the model’s structure and methods are provided elsewhere [9]. The population considered was first structured by HIV transmission risk group and gender: male high risk heterosexuals (HRH), female HRH, men who have sex with men (MSM), male injection drug users (IDUs) and female IDUs. These five subgroups were then further stratified by three race/ethnicities defined as black, Hispanic and all others; where all others are primarily (97%) whites but also included Asians, Pacific Islanders, Alaska natives and American Indians. Studies have demonstrated that those aware of their HIV infection tend to engage in safer sexual behavior with uninfected partners [10], [11], [12], and that transmission rates among those HIV infected and unaware are on average 3.5 times that of those who are infected and aware of their infection [13]. Therefore, each of the 15 population subgroups was divided into three compartments: those susceptible to HIV infection, those who are HIV-positive but undiagnosed, and those diagnosed with HIV.

New infections occur as a result of effective contacts between those who are susceptible and both diagnosed and undiagnosed HIV positive populations. To identify the effective contact rates, first, we use the method suggested by Marks (2006) to estimate the percentage of HIV transmissions from the HIV-positive diagnosed for each of the 15 subgroups [13]. Then, using these data and assumptions about racial and sexual mixing between population subgroups, we establish two 15 by 15 matrices: one for the number of new infections in the susceptible population subgroups that result from contact with the undiagnosed subgroups and one for the number of new infections in the susceptible population subgroups that result from contact with the diagnosed subgroups. Knowing the size of each subgroup compartment, we identify the effective contact rates by solving for them in the incidence equation of a Susceptible-Infected model [14]. This process is detailed elsewhere [9].

The effect of antiretroviral therapy on transmissions is implicit in our model. First, deriving contact rates from incidence and prevalence avoids the need to parse the effects of each factor that contributes to HIV transmission because the number of incident HIV cases is indicative of the effects of all these factors such as number of partners, number of unsafe acts, transmission probabilities, viral load and stage of disease. Also, the percentage of HIV transmissions from the HIV-positive diagnosed assumes that a proportion of those HIV-positive diagnosed have an undetectable viral load and cannot transmit the virus [13].

### Input Data for the Epidemic Model

The estimated size of the HIV-infected population in the US, aged 13–64, for each high-risk population subgroup was based on DHAP’s surveillance data, with three adjustments [15]. First, we adjusted these data for completeness of reporting, which represents cases that have been diagnosed but will never be reported [16]. Second, for the year 2006, HIV estimates are only available for 33 states. Therefore, we extrapolated these HIV estimates to the 50 states and the District of Columbia using the ratio of estimated AIDS prevalence in those 33 states to the AIDS prevalence in the 50 states and the District of Columbia. Third, we adjusted for undiagnosed cases using the proportion of HIV-infected persons who are aware of their infection by race/ethnicity, gender and transmission category [3].

The model also requires estimates of the sizes of the high-risk population subgroups. Our primary source of data for HRH and MSM was cycle 6 (2002) of the National Survey of Family Growth (NSFG) [17]. The size of the HRH population was based on the number of respondents who self-reported any of the following risk behaviors in the past year: STD treatment; HIV positive, IDU or five or more opposite sex partners; sex in exchange for money or drugs; crack cocaine use; and for females, MSM sex partner. Our definition of HRH excludes all MSM and IDUs. The size of the MSM population was based on the estimated number of male respondents who self-reported sex with another male but not illicit drug injection in the past year. Due to underreporting of MSM behavior, we used the upper end of the confidence interval in our estimation. The latest national estimate for the total number of IDUs is 1,543,746 for the year 2002 [18]. We used estimates of injection drug use among black and white residents of US metropolitan areas to break down the national estimate by race/ethnicity [19] and used CDC’s HIV Counseling and Testing System (CTS) to break down the estimates by gender [20].

We analyzed DHAP’s programmatic budget data by line item to estimate the current allocation to each of the 15 population subgroups for testing, and counseling and education intervention; the allocation totals $327 million and we refer to it as the baseline allocation.

The current version of our inputs is based on the most recent data available and we intend to repopulate our model with updated inputs annually.

### The Optimization Model

The optimization model chooses the amounts to allocate each year toward interventions and population subgroups to minimize new infections over a five-year time horizon. The model is informed by the costs and effectiveness of the prevention interventions under consideration. Potential funding amounts can be limited by three factors: the maximum annual budget, upper and lower bounds on the amounts that can be allocated to an intervention and population subgroup, and the minimum and maximum proportions of a population subgroup that can be reached by an intervention. The formulation of the optimization model is provided elsewhere [9].

### HIV Prevention Interventions

We considered the two types of HIV prevention interventions historically funded by CDC: HIV testing, and individual and group-level counseling and education. At the narrowest level, interventions could be targeted any of the susceptible or infected compartments of the population subgroups and at the broadest level interventions could be aimed at the general U.S. population. Individuals who are susceptible to infection are not distinguishable from those who are HIV-infected but undiagnosed and are therefore targeted together.

Typically, intervention costs per person increase with the level of targeting so that more narrowly targeted interventions are costlier per person than broadly targeted ones. When testing is aimed broadly to the general US adult population, we estimated the per person cost of testing based on the cost of opt-out testing in emergency department settings [21], and the cost of a CDC-led expanded testing program. When testing is targeted to high risk populations, we based our cost estimates of testing on the average of several published studies reporting the cost of testing in STD clinic settings and the cost of testing in outreach settings [21], [22]. We identified studies of individual-, and group-based counseling and education interventions with statistically significant positive between-group outcomes and for which sufficient data were reported to conduct a cost assessment and calculate the effect on the participant’s estimated annual acquisition or transmission rate. Estimates of the cost per person and effect size of counseling and education targeted to HIV-positive, diagnosed persons were based on five studies [23], [24], [25], [26], [27]; and these estimates for interventions targeted to HIV-negative, high-risk persons were based on four studies [28], [29], [30]. All costs were adjusted to 2009 US dollars [31]. Data and sources are presented in Table 1.

**Table 1:** Input data.

Parameter	Value	Source
Estimated size of the HIV- population, aged 13–64, in the US (2006)	1,100,000	[3]
Estimated size of the high-risk population, aged 13–64, in the US	21,300,000	[17], [18], [19], [20]
Percentage of HIV positives unaware of their serostatus	21%	[3]
Estimated number of new HIV infections in the US (2006)	48,600	[1], [43]
Proportion of new infections that result from contact with an unaware positive	48%	[13]
Cost of testing per positive in the general US adult population (US$ 2009)	$82	[21]
Cost of testing per negative in the general US adult population (US$ 2009)	$18	[21]
Cost of testing per positive in the high-risk populations (US$ 2009)	$126	[21], [22]
Cost of testing per negative in the high-risk populations (US$ 2009)	$52	[21], [22]
Per person cost of counseling and education interventions for positives (US$ 2009)	$514	[23], [24], [25], [26], [27], [31]
Per person cost of counseling and education interventions for high-risk populations (US$ 2009)	$322	[28], [29], [30], [31]
Effect size of counseling and education interventions for positives	18%	[23], [24], [25], [26], [27]
Effect size of counseling and education interventions for high risk populations	8%	[28], [29], [30]
Reduction in effect size of all counseling and education interventions after 12 months	90%	Assumption

In the epidemic model, testing interventions derive their prevention effects from moving HIV-infected but undiagnosed individuals to the HIV-infected and diagnosed compartment where the effective contacts rates are smaller. Counseling and education interventions derive their prevention effects from removing a portion of those reached from their respective compartments for the duration of effect of the intervention. After 12 months and for the remainder of the time horizon, the effect of counseling and education interventions was assumed to wane by 90% relative to the effect size at 12 months.

### Validation of Model Structure and Inputs

The HIV resource allocation model’s structure and methods were reviewed and validated by four subject matter experts external to the CDC.

We developed a data definition document in which the input data were grouped into 15 categories; for each category, we report the data values, data sources and derivation method. We established an internal validation process where subject matter experts, internal to DHAP, were assigned to each data group and tasked with revising and approving the corresponding data definition document. Once all data definition documents were validated, an internal review panel of 25 members, including but not limited to the assigned experts, was convened. The panel met twice, reviewed all data definition documents and provided revisions and final approval.

Data definition documents were then compiled into six categories: three on the characteristics of the MSM, IDU and HRH populations, and three on intervention program data relating to costs, counseling and education outcomes and maximum penetration rates. For each data category, we established an external peer review committee comprised of two to four consultants outside of CDC. The charge to each committee entailed reviewing the corresponding data definition document, providing a written assessment with references to alternate sources of data where applicable, and participating in a conference call with all respective committee members to finalize the data.

Using this internal and external validation process, we verified all data inputs to the resource allocation model and, where applicable, identified a realistic range of values for use in sensitivity analysis.

We ran the model to optimality under the base case conditions and examined how the recommended optimized allocation differs from the current allocation of funds in terms of populations and interventions.

We developed a univariate sensitivity analysis function in our model to gauge the stability of the model results. This sensitivity analysis function evaluates up to ten variables to be modified, in sequence, according to five user-specified values. We selected key model results and created upper and lower bounds based on a 10 percentage point increase or decrease in the proportion of the budget allocated according to these key model results. For example, if the model suggests allocating 54% of the total budget to testing interventions then the upper and lower bounds were set to 64% and 44%, respectively. We report the cases where one or more of the bounds on the key model results have been violated and provide the related details.

To understand how the model prioritized funding to interventions and populations, we sequentially set the total budget from $100 million to $500 million in increments of $100 million and set the model to optimize for each of those allocations. This scenario can supply valuable information for decision makers because it illustrates how HIV prevention should be prioritized, given limited budgets, to have the greatest impact on the epidemic.

## Results

**Figure 1:**
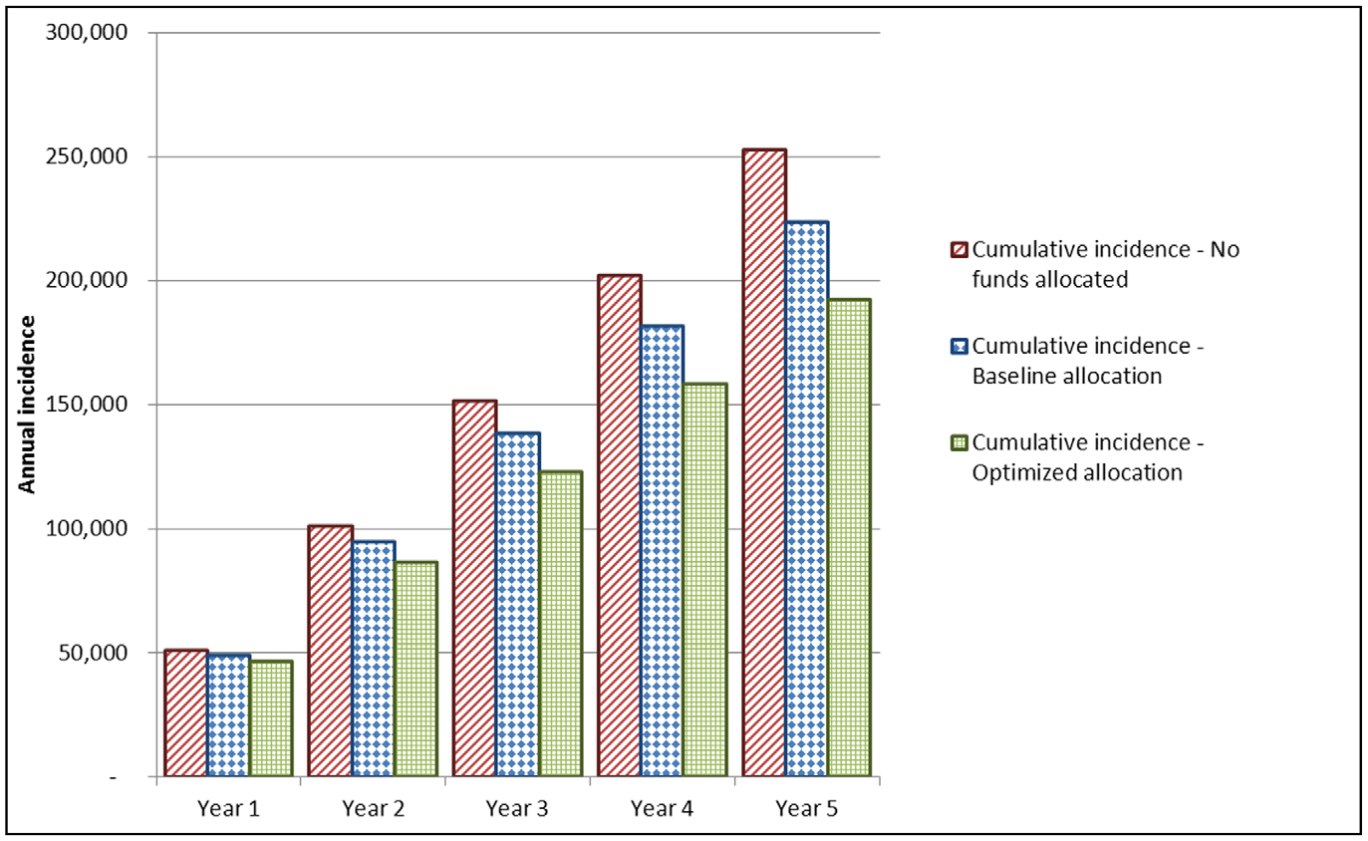
Annual and cumulative number of new HIV infections over 5 years.

The epidemic estimates predicted by the model are presented in Figure 1. The cumulative number of new HIV infections over five years predicted by the model is 192,000 under an optimized allocation of the $327 million budget, 223,000 in the baseline scenario which assumes the current allocation of the $327 million budget and 252,000 assuming no allocation of funds. Thus the baseline allocation averts 13% of new infections when comparing no allocation of funds and the optimized allocation averts 31% of new infections when comparing no allocation of funds. The gap in annual incidence between no allocation and the baseline allocation steadily increased from 4% in the first year of the time horizon to 20% in the fifth and last year. In the first year, the incidence in the optimized allocation represents a 9% reduction relative to the no allocation; because the effects of decreased transmissions accumulate over time, this gap widens and the annual incidence in the fifth year only represents a 48% reduction in the optimized allocation relative to the no allocation. Over the five year horizon, the breakdown of new infections by risk group is approximately 30% HRH, 60% MSM and 10% IDU and is consistent across the optimized, baseline and no allocation strategies.

The HIV resource allocation model results are presented in Table 2. Key differences between the baseline and the optimized allocation recommended by the model include: first, 29% of the total budget of $327 million was allocated to the general US adult population at the baseline, while the optimized scenario allocated the entire budget to the MSM, IDU and heterosexual high risk populations, and no funds to the general US adult population. Second, 49% of the total budget was allocated to testing and 51% to counseling and education at the baseline, while the optimized scenario allocated 61% and 39% of the budget to testing and counseling and education interventions, respectively. Also, 11% of the counseling and education budget was targeted to counseling and education for diagnosed positives at the baseline and that percentage rose to 100% according to the model’s optimized allocation. Lastly, the allocation to MSM increases from 23% to 51% of the total budget between the baseline and the optimized scenario, and the allocation to at-risk blacks and Hispanics increases from 49% to 65% of the total budget. In the optimized scenario, funds for MSM are targeted to testing black and Hispanic MSM and to counseling and education for diagnosed, positive MSM.

**Table 2:** Allocated proportion of budget.

	Baseline	Model
Allocation by intervention		
Counseling and education	51%	39%
Testing	49%	61%
Allocation by intervention and risk group		
Testing the general population	23%	0%
Testing MSM	6%	36%
Testing IDUs	3%	11%
Testing HRH	16%	14%
Counseling and education for the general population	6%	0%
Counseling and education for MSM	17%	15%
Counseling and education for IDUs	8%	0%
Counseling and education for HRH	20%	24%
Allocation by intervention and race/ethnicity a		
Testing blacks	12%	21%
Testing Hispanics	5%	9%
Testing others	9%	31%
Counseling and education for blacks	20%	15%
Counseling and education for Hispanics	12%	20%
Counseling and education for others	13%	4%
Allocation to counseling and education by serostatus		
Counseling and education for diagnosed positives	11%	100%
Counseling and education for susceptibles^b^	89%	0%

a May not total 100%. Includes funds reaching to the 3 risk groups only, not the general population.

b Undiagnosed positive persons are not distinguishable from susceptible persons so they are targeted together.

### Sensitivity Analyses

In order to assess the stability of the model results, we conducted more than 100 one-way sensitivity analysis scenarios on more than 20 model variables. Below, we present only scenarios where variations in the parameter values altered any of the key results presented in Table 2 by more than ten percentage points.

Results appeared most sensitive to variations in the cost of testing, the cost and outcome of counseling and education interventions and the size of the MSM population. The model’s optimized allocation to testing MSM increased by more than ten percentage points when the cost of testing increases beyond $150 per negative and $225 per positive because testing MSM remains a priority in spite of the cost increase and therefore the allocation to testing MSM is proportionally greater.

The effects observed when either reducing the cost or increasing the outcome of counseling and education programs are similar because they improve the cost-effectiveness of counseling and education. Under these circumstances, the overall allocation to counseling and education programs increases, and therefore the allocation to testing is reduced; also, the proportional allocation to targeted MSM for both intervention types increases thereby confirming them as a high priority risk group.

As the size of the MSM population in the model increases, the proportion of the allocation to testing that is targeted to MSM increases by more than ten percentage points, indicating a sustained focus on MSM.

### Incremental Budget Constraint Scenario

**Figure 2:**
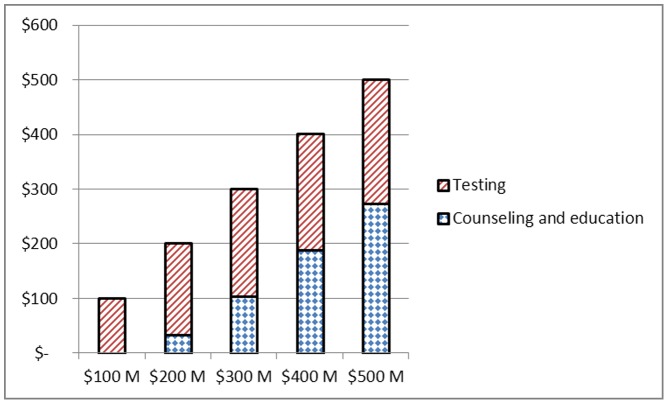
Model allocation to intervention types by budget amount.

**Figure 3:**
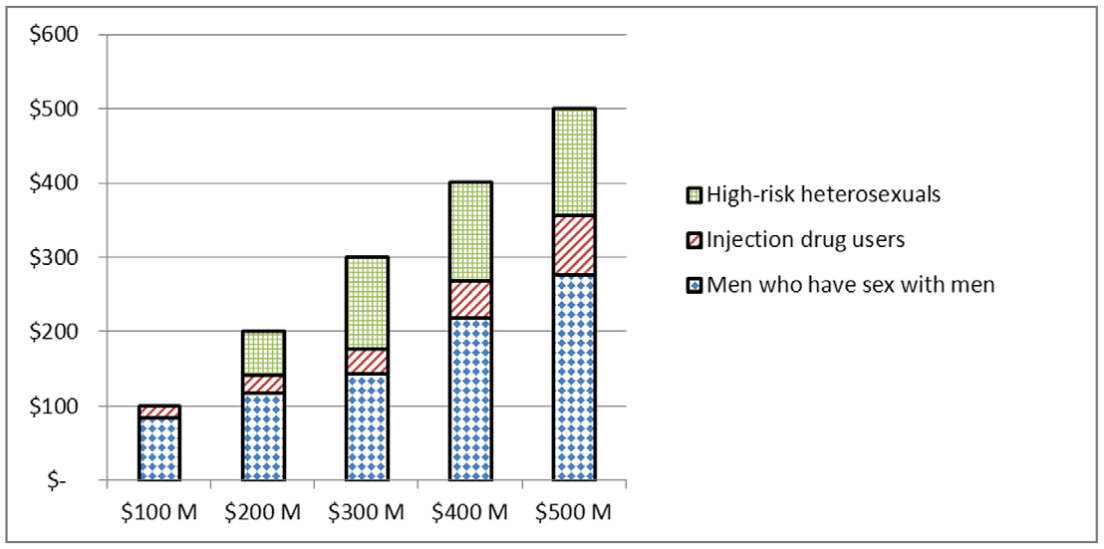
Model allocation to risk groups by budget amount.

We evaluate five budget scenarios where the total budget is set between $100 million and $500 million in increments of $100 million. Figure 2 displays the allocation to testing and counseling and education interventions for each budget scenario. At $100 million, the budget is allocated to testing only but as the budget increases, more funds are allocated to counseling and education interventions and at a budget of $500 million more funds are allocated to counseling and education interventions than to testing. Figure 3 presents the allocation by risk group. At $100 million, 84% of the budget is allocated to MSM and the remainder to IDUs; as the budget increases, more funds are allocated to all three risk groups. At a budget of $500 million the proportion of funds allocated to MSM, IDUs and HRH is 55%, 16% and 29% respectively. Table 3 presents new infections over the five-year horizon as the annual budget increases. The marginal infections averted decrease from 38,506 to 5,906 and represent the reduction in HIV incidence for each additional $100 million made available in the annual budget. The total number of infections averted relative to no investment is also presented in Table 3 along with the resulting cost per infection averted, medical costs averted and an estimate of the cost-savings incurred which are increasing from $13 billion to $23 billion. The medical costs are based on the lifetime treatment cost of an HIV infection estimated at $367,000 (US$ 2009) [32].

**Table 3:** New infections and costs by budget amount given model allocation.

Annualbudget	New infectionsover 5 years	Marginal infectionsaverted^a^	Total infectionsaverted^b^	Total cost perinfection averted^c^	Total medicalcosts averted	Total costsavings
0	252,381	–	–	–	–	–
$100 million	213,874	38,506	38,506	12,985	$ 14,131,805,904	$ 13,631,805,904
$200 million	202,529	11,346	49,852	20,059	$ 18,295,608,351	$ 17,295,608,351
$300 million	194,051	8,477	58,329	25,716	$ 21,406,800,373	$ 19,906,800,373
$400 million	187,334	6,718	65,047	30,747	$ 23,872,126,976	$ 21,872,126,976
$500 million	181,427	5,906	70,953	35,235	$ 26,039,804,094	$ 23,539,804,094

a Refers to the number of infections averted relative to the next annual budget increment.

b Refers to the number of infections averted relative to no investment of funds.

c Calculated as the total budget over 5 years divided by the total infections averted.

## Discussion

Our model estimates reductions in new HIV infections associated with current and optimized HIV prevention expenditures and results in three main recommendations. First, the allocation to testing interventions should increase and further target MSM and IDUs. Second, counseling and education interventions ought to provide a greater focus on HIV-positive persons. And lastly, more funds should be allocated to those at high risk rather than the general population. As expected, annual incidence is highest given no allocation of funds and minimized under the model’s optimized allocation of the budget.

Relative to no investment of funds, the baseline and optimal allocation scenarios avert 29,035 and 60,271 HIV infections, respectively. Given the programmatic annual budget of $327 million, this implies a cost of $56,311 per infection averted for the baseline as compared to no allocation and $27,128 per infection averted for the optimal allocation as compared to no allocation. Therefore, both the current baseline and the optimal allocation of funds can be considered cost-saving when compared to the HIV lifetime treatment costs. The baseline and optimal scenarios allocate the same budget amount so a cost per infection averted relating both scenarios cannot be inferred. The undiscounted expenditures total $1.6 billion while the total medical cost of treating the infections should they not be averted would be $11 billion under the current allocation strategy and $22 billion under the optimized allocation. In the incremental budget scenario, the marginal infections averted decreases and the cost per infection averted increases as the annual budget increases reflecting the decreased rate at which new infections are averted given additional funding. At the lowest budget level, funds are spent on the most cost-effective interventions and target groups, as the budget increases and the maximum capacity constraints are reached for those most cost-effective interventions and target groups, the additional funds are allocated to less cost-effective targets thereby increasing the cost per infection averted. Nonetheless, even at $500 million per year, the lifetime HIV treatment cost of $367,000 [32] exceeds the estimated cost per infection averted by $332,000 indicating a cost-saving level of investment.

Given the many inputs and outputs to our model, sensitivity analysis is not straightforward. Our objective in sensitivity analysis was to evaluate whether the main model recommendations are upheld throughout reasonable variations in the input data. Of over 100 sensitivity analysis scenarios conducted, only 9 scenarios altered the key results presented in Table 2 by more than ten percentage points. However, those scenarios tended to reinforce the model’s recommended focus on MSM. The budget constraint scenario highlights the critical importance of testing, targeting high-risk groups and diagnosed positives. The classic epidemic control theory of focusing on high-transmission core groups [33] endorses our results.

Our model is a simplified representation of the actual allocation process of DHAP’s HIV prevention resources and thus translating the model’s output into practice can be difficult. The model assumed that all members of high-risk population subgroups are reachable and can be perfectly targeted with interventions. This simplifying assumption may lead to an overestimation of the model’s impact because some target population members cannot be easily reached (and may not even know that they or their partner are at risk for HIV) and programs for these populations will have to contact some persons who are not target population members in order to reach those who are most at-risk. Thus, the programmatic efficiency assumed in the model cannot be achieved in real-world programs. Nonetheless, our model provides insights on causal relationships and helps us to identify areas where prevention programs can have the most impact.

Results of the HPTN052 randomized control trial among serodiscordant couples indicated that antiretroviral therapy reduces incidence of HIV transmission to the negative partner by 89% [8]. Our model does not explicitly consider antiretroviral treatment costs or the variation in HIV transmission rates associated with viral load suppression and stage of disease. However, early diagnosis and treatment reduces HIV transmission so the benefits of HIV diagnoses may be underestimated in this analysis and an even greater focus on testing than suggested by our model results may be warranted.

We made linear assumptions on scalability in the intervention cost and outcome functions of the model; and the shape of such functions may make a difference in the optimal allocation of funds [34], [35]. Economic evaluations (such as cost-effectiveness analysis) of HIV prevention programs typically consider one funding level and their results do not inform on the additional benefit to be gained (or lost) through increasing or decreasing the investment [36], [37], [38]. While our model structure could support more complex functions, the true shape of such functions is not known and no reliable data exist to support them.

The per-person intervention costs in our model were largely derived from microcosting methods where all cost items relating to an intervention are detailed and cumulated. In contrast with program budget methods where the overall allocated budget is simply divided by the number of persons served, microcosting leads to an underestimate of per-person cost [39]. In actuality, federally allocated funds are not typically spent completely on the direct costs of implementing programs; some portion may be spent on the indirect costs of organizations that implement the interventions and other entities involved in getting funds to those organizations. For example, DHAP allocates funds to state health departments who in turn fund local health jurisdictions, who may fund community-based organizations to deliver an intervention. Microcosting does not include the additional funds spent channeling the resources through these levels to the intended intervention. Though the method we applied may overestimate the numbers of persons that can be reached given the overall budget constraint, the budget constraint scenario presented mitigates this limitation. If the overage associated with indirect costs is 60%, then the available budget for program spending would be about $200 million, and that scenario echoes the main conclusions of the model.

As with most studies, input data are often being revised and updated. All data inputs were reviewed internally by a panel of 25 persons within the CDC, data were then validated through an external peer review process involving by 18 experts. We plan to publish updates of this analysis, including the effects of newly implemented programs, including the Expanded HIV Testing Initiative for populations disproportionately affected by HIV, primarily African Americans.

Models cannot encompass all the dynamic, complex and often qualitative realities of HIV resource allocation or be expected to provide prescriptive results [40]. Resource allocation models for the control of infectious diseases, such as the one presented here, are intended to provide guidance on improving the allocation of funds; they are not designed to provide accurate epidemic projections. Such models are typically comprised of an optimization component that sequentially supplies allocation scenarios to an epidemic component which projects infections until the optimal allocation is found. This process can easily reach millions of iterations and depends on the number of variables and constraints; therefore, the epidemic component must be kept simple and efficient. More comprehensive epidemic models that are neither bound to an optimization engine nor anchored to a time horizon that reflects a budget cycle can be designed to measure epidemic growth more precisely over time. Given the aforementioned considerations, our model results are not intended to make epidemic projections over time, though their interpretation as a measure of the difference in impact between scenarios is robust.

Our results have, in part, provided the impetus for other programs, such as the Expanded HIV Testing Initiative. A recent analysis of the Initiative suggests that the program achieved a return of $1.95 for every dollar invested by CDC [41]. Lastly, according to the National HIV/AIDS Strategy (NHAS), intensifying prevention efforts for MSM, IDUs, black and Hispanic men and women, and increasing the percentage of people living with HIV who know their serostatus is required to reduce annual HIV incidence [42]. The main conclusions of the HIV resource allocation model serve to substantiate and provide rational economic evidence for the NHAS goals. Decision makers responsible for the allocation of DHAP’s programmatic funds report that the national HIV resource allocation model, along with program and other data, provides valuable guidance to target resources and improve the impact of HIV prevention efforts in the United States.
